# Microbiological quality of some packed and unpacked bread products in Alexandria, Egypt

**DOI:** 10.1186/s42506-023-00141-9

**Published:** 2023-08-16

**Authors:** Manal A. Ali, Mona H. Hashish, Marwa M. Fekry

**Affiliations:** 1 Medical Physiology Department, Alexandria Faculty of Medicine, Alexandria University, Alexandria, Egypt; 2 Department of Microbiology, High Institute of Public Health, Alexandria University, 165 El-Horreya Avenue, Alexandria, Egypt

**Keywords:** Bakery products, Microbiological quality, Packed bread, Unpacked bread

## Abstract

**Background:**

Bakery products are important food snacks consumed by people of all ages and economic groups. The growth of unwanted microorganisms that deteriorate products such as bacteria, moulds, and fungi in these foodstuffs may offer risks to consumers’ health and generate considerable economic losses. This work aimed to assess the microbiological quality of some packed and unpacked bread products in Alexandria, Egypt.

**Methods:**

This cross-sectional comparative study involved 168 local and branded bakery products that were collected randomly from 2 districts in Alexandria. Hygienic practices such as covering of the bread and wearing gloves during handling were observed and recorded. All bread samples were tested to determine the total plate count (TPC), presence/absence of *Staphylococcus aureus* (*S. aureus*), total yeasts and moulds in CFU/g and total coliform count (TC) in MPN/g.

**Results:**

The mean of the total yeasts and moulds and TC in the packed bread was lower than that of the unpacked bread (3.40 × 10^3^ CFU/g and 3.25 MPN/g *versus* 6.37 × 10^3^ CFU/g and 31.61 MPN/g, respectively). However, the mean of TPC in the packed bread was higher than that of the unpacked bread (1.39 × 10^6^ *versus* 2.07 × 10^5^ CFU/g, respectively). The mean TPC, total yeasts and moulds and TC was higher in the studied flatbread than Fino bread and toast (3.4 × 10^6^, 1.14 × 10^4^ CFU/g and 24.6 MPN/g, respectively). The presence of *S. aureus* was higher in flat, unpacked bread, bread displayed outside the shop and handled without gloves.

**Conclusion:**

Bread produced by local bakeries showed lower standards in packaging and microbial quality. Better manufacturing, packaging, storage, and handling initiatives should be introduced to avoid related food safety concerns in the future. The formal authorities should define and clarify standards and rules on bread safety.

## Introduction

Bakery products are among the most important basic foods consumed daily by people throughout the world [1]. They are universally accepted as a very convenient form of food that has desirability to all populations rich and poor, rural and urban [2]. In several countries in the world, up to 50% of the total required calories are supplied by bread alone.

Egypt is one of the largest consumers of bread and bakery products in the world, ranking sixth in 2016 [3]. The Egyptian market is dominated by two types of bread, the frontrunner is flatbread (Baladi bread); and French bread (Fino) which is popular among consumers due to its fine texture, fluffy crumb, high specific volume, and slight buttery-sweet taste [4].

Bread and other bakery products are subjected to various spoilage problems; physical, chemical and microbial; the latter being the most serious one. The most common microbial contaminants of bread are *Bacillus spp.* and various moulds. Food safety has become very important for governments, food manufacturers as well as consumers. Like other food products, microbiological hazards have also become a public health safety concern for bakery products [5, 6].

Spoilage of bread and other bakery products can manifest as inanimate physical and chemical spoilage with moisture loss or rancidity, or in the form of animate spoilage due to growth of moulds or bacteria. The spoilage potential of bakery products is dependent on their acidity and water activity, with high moisture and low acidity products being the most susceptible to microbiological spoilage [7].

Contamination occurs, after the baking process, during the various production steps such as cooling, slicing (unhygienic handling), transportation and packaging as well as storage. Bakery products within the production and storage chain are contaminated with moulds, yeasts, and occasionally bacteria [2]. Unhygienic packaging conditions and unsatisfactory storage of bakery products can worsen their microbial quality. Therefore, it is important to monitor the microbial quality of bread products according to international standards.

Despite the beneficial role of specific microorganisms in the production of bakery products (to enhance consistency and improve flavouring), some of them may cause spoilage of bakery products. In addition to bacteria, moulds and yeasts are common causes of such spoilage. Freshly baked products are sterile and do not contain viable microorganisms but can become contaminated when exposed to air and surfaces [8].

Unsafe food containing harmful bacteria, viruses, parasites or chemical substances causes more than 200 diseases, ranging from diarrhea to cancers. It also creates a vicious cycle of disease and malnutrition, particularly affecting infants, young children, elderly, and the sick. The 2019 World Bank report on the economic burden of the foodborne diseases indicated very high total productivity loss and annual cost of treating foodborne illnesses in low- and middle-income countries, and the [9]. In recent years, foodborne illness outbreaks linked to wheat flour have been reported in many countries of the world, including Australia, Europe, and the United States of America. Bacterial pathogens like Shiga toxin–producing *Escherichia coli* (STEC), *Salmonella* species, *Bacillus cereus*, and other spoilage microorganisms were identified as hazardous [10]. Contamination of bread products by fungi is a cause for concern because it can reduce the product shelf-life generating economic losses and consumers dissatisfaction. Moulds and fungi also produce different types of “mycotoxin”, which have been linked to many different diseases including cancers, diabetes, and internal organ damage [11].

The maximum permissible limits in baked products (cake, bread, and biscuits) were set for total plate count (TPC) to be < 10^5^ cfu/g, for yeasts and moulds < 10^4^ cfu/g, for total coliform TC < 200 MPN/g and absence of *E. coli* [12]. To our knowledge, a few studies were performed on the microbiological status of bread and flour in Egypt. Therefore, it is of public health importance to assess the microbiological quality of bread in Egyptian markets. The current study was conducted to assess the microbiological quality of some packed and unpacked bread in two districts in Alexandria, Egypt. This was achieved through the determination of the TPC, total yeasts and moulds in colony forming unit/gram (CFU/g) and total coliform (TC) in most probable number in grams (MPN/g) as well as the presence/absence of *staphylococcus aureus (S. aureus)* in the studied bread samples collected from both districts.

## Methods

### Study design

The present cross-sectional comparative study was carried out during the period from November 2021 through March 2022. It involved 168 bread samples collected randomly from 2 districts in Alexandria, where 84 samples were packed, and another 84 samples were unpacked. Bread samples were purchased from sellers and handled in the same way as any client.

### Sample

The minimum required sample size was calculated using G*Power software, assuming a moderate effect size in bacterial count with twelve groups (three types of bread: Baladi bread, Fino bread, and toast; two types of packaging: packed and unpacked; and two types of wheat: white flour and whole wheat flour) at an alpha error of 0.05 and a power of 80%. The minimum required sample size was found to be 158 (approximately 14 from each group).

Two-stage cluster sampling was used. Two districts were selected randomly from Alexandria Districts (Al Montaza and Sharq): In each selected district, one central location and one direction were chosen randomly to start with, all bakery shops in that direction were included and different types of bread were purchased. Consecutive samples were collected until the required sample size was fulfilled.

### Data collection

A data sheet was filled for each bread sample including the type of sample, site, date and time of collection, expiry date and duration of storage, location of storage, whether bread was covered or not and whether gloves were worn by the seller. The study protocol was approved by the Ethics Committee at the High Institute of Public Health (HIPH).

### Laboratory procedures

Twenty-five grams of each bread sample was cut and mixed with 225 ml of sterile peptone water (PW) in a sterile stomacher plastic bag in the stomacher for 1–2 min to be completely homogenized. This constituted the first dilution 10^–1^ from the original sample [13]. All bread samples were subjected to the following tests: determination of TPC, total yeasts and moulds, TC in MPN/g as well as isolation and identification of *S. aureus*. All the microbiological procedures were performed according to the methods described in Laboratory Methods in Food Microbiology [13].

- I. Determination of the total plate count (TPC) using pour plate method
  Tenfold dilutions were prepared from the sample homogenate in the diluent buffer of PW (1 ml of homogenate + 9 ml of diluent buffer) to get 10^–2^ and 10^–3^ dilutions. One ml of each dilution was pipetted into a sterile petri dish (duplicate plates) under complete aseptic conditions. Twenty ml of melted sterile plate count agar (45**°**C) was then poured into each Petri dish and the contents were mixed thoroughly by rotating the plate several times, clockwise and then anti-clockwise. When the media had solidified, the plates were inverted and incubated at 37**°**C for 48 h. Control plates were included to ensure the sterility of the plates. Plates having colony counts between 25 and 250 colonies were chosen and counted using the Quebec counter. The average number of colonies/plate was multiplied by the dilution factor and recorded as CFU/g.
- II. Enumeration of total coliforms (TC) by multiple tube dilution method
  A presumptive test for TC was performed using three tubes of lauryl sulphate tryptose (LST) broth. Nine ml in each tube were inoculated with one ml of the previously prepared 1:10, 1:100 and 1:1000 dilutions. Tubes containing inverted Durham’s tubes were used for gas detection and were incubated aerobically at 37°C for 24–48 h. All LST tubes showing both turbidity and gas within 48 h were recorded as positive presumptive tubes and the Most Probable Number (MPN) was obtained from MPN food tables for the recorded 3 tube dilutions. Results were recorded as the presumptive MPN of coliform bacteria per gm.
  A confirmed test was done for all positive presumptive tubes. Three loopfuls of presumptive positive tubes were inoculated into each Brilliant Green Lactose Bile Broth (BGLBB). All tubes were shaken on a vortex mixer and incubated at 35- 37°C for 24–48 h. Tubes showing turbidity and gas were recorded as positive confirmed tests for TC.
  Positive confirmed BGLBB tubes were streaked on eosin methylene blue (EMB) plates for 24 h at 37°C. The typical nucleated (dark-centered) colonies with or without sheen were subjected to Gram staining. The typical microscopic appearance of gram-negative short non-spore forming rods identifies the presence of TC.
- III. Isolation and identification of S. aureus by surface plating procedure
  One-tenth ml of the prepared dilution was plated onto each of two plates of Baird-Parker agar with added egg yolk tellurite enrichment. The inoculum was spread over the surface of the agar using a sterile streaking rod. The plates were retained in an upright position until the inoculum was absorbed by the medium. Inverted plates were incubated aerobically for 24–48 h at 35–37°C. Gray to black colonies surrounded by an opaque area were picked and Gram stained. Typical *S. aureus* colonies were gram-positive cocci in clusters. The catalase test was used to differentiate between catalase-positive organisms like *staphylococci* from catalase-negative species like *streptococci*. The coagulase test was used to identify coagulase-positive *S. aureus*.

### Statistical analysis

Data was fed to the computer and analyzed using IBM SPSS software package version 23.0***.*** Qualitative data were described using numbers and percentages.

The Kolmogorov- Smirnov test was used to verify the normality of distribution. Quantitative data were described using range (minimum and maximum) and median. The significance of the obtained results was judged at the 5% level. The Chi-square test was used for categorical variables, to compare different groups. Monte Carlo test significance was used for C*R (> 2*2) tables where the expected count in more than 25% of cells was less than 5. Mann–Whitney test was used for abnormally distributed quantitative variables, to compare two studied groups. Kruskall Wallis test was used for abnormally distributed quantitative variables, to compare more than two studied groups [14].

## Results

In both districts, gloves were mostly used in handling the white and bran toast, while white and bran flatbread were the most types handled without gloves. In Al Montaza District, Fino bread and toast were the most covered bread types, while flat bran bread was the most uncovered bread type. It was also noted that most bread types displayed inside the shop were Fino and toast. The majority of bread samples displayed outside the shop were white and bran flatbread. *S. aureus* was detected in 21.7% (18 out of 83) of bread in Al Montaza District and in 11.8% (10 out of 85) of bread in Sharq District with no statistically significant difference between the two districts as regards the presence of *S. aureus* (*p*-value = 0.084). The mean TPC and total yeasts and moulds in bread collected from Al Montaza District was significantly higher than that of bread collected from Sharq District (1.4 × 10^6^ *versus* 1.3 × 10^5^ CFU/g and 9.28 × 10^3^ *versus* 5.97 × 10^2^ CFU/g, respectively) with *p*-value < 0.001. However, the mean TC in Al Montaza bread and Sharq bread was comparable (17.4 *versus* 17.4 MPN/g, respectively) with no statistically significant difference (*p*-value = 0.577) (Table 1).

**Table 1:** Distribution of *S. aureus*, TC, TPC and total yeasts and moulds in the bread according to Alexandria districts, 2021–2022

	**District**				**Test of significance**
	**Al Montaza (*n* = 83)**		**Sharq (*n* = 85)**		
***S. aureus* (CFU/g)**	**N**	**%**	**N**	**%**	$x$ ^2^ = 2.977 *p* = 0.084
Present	18	21.7	10	11.8	
Absent	65	78.3	75	88.2	
**Total plate count (CFU/g)**					
Mean ± SD	1.4 × 10^6^ ± 8.5 × 10^6^		1.3 × 10^5^ ± 3.3 × 10^5^		U = 2252.0 *p* < 0.001*
Median (IQR)	1.64 × 10^4^ (56.98 × 10^4^)		70.0 (1.35 × 10^4^)		
Min–Max	0.0–7.7 × 10^7^		0.0–2.2 × 10^6^		
**Total yeasts and moulds (CFU/g)**					
Mean ± SD	9.28 × 10^3^ ± 2.95 × 10^3^		5.97 × 10^2^ ± 2.46 × 10^2^		U = 2259.5 *p* < 0.001*
Median (IQR)	1.0 × 10^2^ (2 × 10^3^)		0.0 (100.0)		
Min–Max	0.0–15 × 10^4^		0.0–2 × 10^4^		
**Total coliforms (MPN/g)**					
Mean ± SD	17.4 ± 120.6		17.4 ± 119.2		U = 3606.0 *p* = 0.577
Median (IQR)	3.0 (0.0)		3.0 (0.0)		
Min–Max	3.0–1.1 × 10^3^		3.0–1.1 × 10^3^		

p* significant at level < 0.05

*IQR* Interquartile range, $x$^2^ Chi square test, *U* Mann–Whitney test; nonparametric used to compare quantitative variables in two groups, *TPC* Total plate count, *S. aureus Staphylococcus aureus*, *TC* Total coliform count, *CFU/g* Colony forming unit/gm, *MPN/g* Most probable number/gm, *SD* Standard deviation

The mean total count of yeasts and moulds and the mean TC of the packed bread were both lower than that of the unpacked bread (3.40 × 10^3^ CFU/g and 3.25 MPN/g *versus* 6.37 × 10^3^ CFU/g and 31.61 MPN/g, respectively) with no statistically significant difference. (*p*-value = 0.145 and 0.065, respectively). However, the mean TPC in the packed bread was higher than that of the unpacked bread (1.39 × 10^6^ *versus* 2.07 × 10^5^ CFU/g, respectively) with no statistically significant difference (*p*-value = 0.923) (Table 2).

**Table 2:** Distribution of TPC, TC and total yeasts and moulds in packed and unpacked bread samples, Alexandria, 2021–2022

	**Packaging**		
	**Packed** ***n* = 84**	**Unpacked** ***n* = 84**	***p* value**
**Total plate count (CFU/g)**			
Mean ± SD	1.39 × 10^6^ ± 8.54 × 10^6^	2.07 × 10^5^ ± 4.09 × 10^5^	*p* = 0.923 U = 3558.0
Median **(**IQR**)**	1.34 × 10^3^	6.9 × 10^2^	
Min–Max	0.0—7.74 × 10^7^	0.0—2.22 × 10^6^	
**Total yeasts and moulds (CFU/g)**			
Mean ± SD	3.40 × 10^3^ ± 1.73 × 10^4^	6.37 × 10^3^ ± 2.45 × 10^4^	*p* = 0.145 U = 3938.5
Median **(**IQR**)**	0.0 (350.0)	0.0 (725.0)	
Min–Max	0.0–15 × 10^4^	0.0–15 × 10^4^	
**Total coliforms (MPN/g)**			
Mean ± SD	3.25** ± **2.18	31.61** ± **168.42	*p* = 0.065 U = 3737.5
Median **(**IQR**)**	3.0 **(**0.0**)**	3.0 **(**0.0**)**	
Min–Max	3.0–23.0	3.0–1.1 × 10^3^	

p* significant at level < 0.05

*IQR *Interquartile range, *TPC* Total plate count, *TC* Total coliform count, *CFU/g* Colony forming unit/gm, *MPN/g* Most probable number/gm, *SD* Standard deviation

It was also noted that the mean TPC in the flatbread was significantly higher than that of the Fino bread and toast (3.4 × 10^6^ *versus* 5.48 × 10^5^ and 1.4 × 10^6^ CFU/g, respectively) (*p*-value < 0.001). In addition, the mean total yeasts and moulds and TC in the flatbread was higher than that of the Fino bread and toast (1.14 × 10^4^ CFU/g and 24.6 MPN/g *versus* 1.56 × 10^3^ CFU/g, 4.74 MPN/g and 1.93 × 10^2^ CFU/g, 22.3 MPN/g, respectively) with no statistically significant difference between the three bread types (*p*-value = 0.06 and 0.493, respectively) (Table 3).

**Table 3:** Distribution of TPC, TC and total yeasts and moulds in the bread samples (flat, Fino, and toast), Alexandria, 2021–2022

	**Type of bread**			
	**Flat** ***n* = 56**	**Fino** ***n* = 54**	**Toast** ***n* = 58**	***p* value of Kruskal Wallis test**
**Total plate count (CFU/g)**				
Mean ± SD	3.4 × 10^6^ ± 7.6 × 10^6^	5.48 × 10^5^ ± 1.90 × 10^6^	1.4 × 10^6^ ± 1.0 × 10^6^	*p* < 0.001* KW = 16.953
Median (IQR)	1.8 × 10^4^ (5.69 × 10^5^)	6.15 × 10^2^ (5.70 × 10^5^)	0.0 (1.01 × 10^4^)	
Min–Max	0.0–5.36 × 10^7^	0.0–1.14 × 10^7^	0.0–7.74 × 10^7^	
**Total yeasts and moulds (CFU/g)**				
Mean ± SD	1.14 × 10^4^ ± 3.49 × 10^4^	1.56 × 10^3^ ± 7.09 × 10^3^	1.93 × 10^2^ ± 5.77 × 10^2^	*p* = 0.060 KW = 5.641
Median (IQR)	1.0 × 10^2^ (1.0 × 10^3^)	0.0(1.0 × 10^2^)	0.0 (4.25 × 10^2^)	
Min–Max	0.0 -15 × 10^4^	0.0 – 5 × 10^4^	0.0 – 4 × 10^3^	
**Total coliforms (MPN/g)**				
Mean ± SD	24.6 ± 146.8	4.74 ± 12.25	22.3 ± 144.0	*p* = 0.493 KW = 1.414
Median (IQR)	3.0 (0.0)	3.0 (0.0)	3.0 (0.0)	
Min–Max	3.0–1.1 × 10^2^	1.0–93.0	3.0–1.1 × 10^3^	

p* significant at level < 0.05

*KW* Kruskal Wallis test: nonparametric used to compare quantitative variables in more than two groups. *IQR* Interquartile range, *TPC* Total plate count, *TC* Total coliform count, *CFU/g* Colony forming unit/gm, *MPN/g* Most probable number/gm, *SD* Standard deviation

The mean TPC and the total yeasts and moulds in bread displayed outside the shop were significantly higher than that of the bread displayed inside the shop (1.94 × 10^6^ and 1.12 × 10^4^ *versus* 1.98 × 10^5^ and 1.53 × 10^3^ CFU/g, respectively) with *p*-value < 0.001 for each. Also, the mean TC in bread displayed outside the shop was higher than that of bread displayed inside the shop (23.8 and 14.0 MPN/g, respectively) with no statistically significant difference (*p*-value = 0.558) (Table 4).

**Table 4:** Distribution of TPC, TC and total yeasts and moulds according to the display of bread (inside and outside the shop), Alexandria, 2021–2022

	**Display of bread**		
	**Inside the shop** ***N* = 106**	**Outside the shop** ***N* = 58**	***p*-value**
**Total plate count (CFU/g)**			
Mean ± SD	1.98 × 10^5^ ± 6.1 × 10^5^	1.94 × 10^6^ ± 1.0 × 10^7^	*p* < 0.001* U = 4490.5
Median (IQR)	1.65 × 10^2^ (3.72 × 10^4^)	6.1 × 10^4^ (5.69 × 10^5^)	
Min–Max	0.0–5.3 × 10^6^	0.0–7.7 × 10^7^	
**Total yeasts and moulds (CFU/g)**			
Mean ± SD	1.53 × 10^3^ ± 5.66 × 10^3^	1.12 × 10^4^ ± 3.46 × 10^4^	*p* = 0.001* U = 4046.5
Median (IQR)	0.0 (1.25 × 10^2^)	1.0 × 10^2^ (1.0 × 10^3^)	
Min–Max	0.0–5.0 × 10^4^	0.0–1.5 × 10^5^	
**Total coliforms (MPN/g)**			
Mean ± SD	14.0 ± 104.8	23.8 ± 144.3	*p* = 0.558 U = 3268.5
Median (IQR)	3.0 (0.0)	3.0 (0.0)	
Min–Max	3.0–1.1 × 10^3^	3.0–1.1 × 10^3^	

p* significant at level < 0.05

*U* Mann–Whitney test; nonparametric used to compare quantitative variables in two groups, *IQR* Interquartile range, *TPC* Total plate count, *TC* Total coliform count, *CFU/g* Colony forming unit/gm, *MPN/g* Most probable number/gm, *SD* Standard deviation

In addition, the mean TC in the uncovered unpacked bread was significantly higher than that of the covered unpacked bread (99.1 and 6.2 MPN/g, respectively) with *p*-value = 0.045. Also, the mean TPC and the total count of yeasts and moulds in the uncovered unpacked bread were higher than that of the covered unpacked bread (2.3 × 10^5^, 6.5 × 10^3^ and 1.5 × 10^5^, 6.3 × 10^3^ CFU/g, respectively) with no statistically significant difference (*p*-value = 0.679 and 0.794, respectively). It was also found that using gloves to handle the unpacked bread was accompanied by a lower TPC and counts of yeasts and moulds than when no gloves were used (mean TPC and counts of yeasts and moulds were 1.8 × 10^5^ and 3.43 × 10^3^ *versus* 2.2 × 10^5^ and 8.27 × 10^3^ CFU/g, respectively. There was no statistically significant difference between using and not using gloves in bread handling as regards TPC and total yeasts and moulds (*p*-value = 0.576 and 0.558, respectively). As regards TC, their mean when using gloves for the unpacked bread was significantly lower than that without using gloves in bread handling (3.0 and 50.1 MPN/g, respectively) with *p*-value = 0.011 (Table 5).

**Table 5:** Distribution of TPC, TC and total yeasts and moulds according to coverage and gloves usage of unpacked bread, Alexandria, 2021–2022

	**Coverage (N = 84)**			**Glove Usage (N = 84)**		
	**Covered** ***N* = 61**	**Uncovered** ***N* = 23**	***p*-value**	**Used** ***N* = 33**	**Not** ***N* = 51**	***p-*value**
**Total plate count (CFU/g)**						
Mean ± SD	1.5 × 10^5^ ± 2.5 × 10^5^	2.3 × 10^5^** ± **4.0 × 10^5^	*p* = 0.679 U = 742.0	1.8 × 10^5^ ± 3.8 × 10^5^	2.2 × 10^5^ ± 4.2 × 10^5^	*p* = 0.576 U = 901.5
Median (IQR)	6.8 × 10^2^ (5.70 × 10^5^)	3.7 × 10^3^ (5.69 × 10^5^)		5.50 × 10^2^ (1.27 × 10^5^)	2.0 × 10^3^ (5.70 × 10^5^)	
Min–Max	0.0–2.2 × 10^6^	0.0–5.7 × 10^6^		0.0–1.7 × 10^6^	0.0–2.2 × 10^6^	
**Total yeasts and moulds (CFU/g)**						
Mean ± SD	6.3 × 10^3^ ± 2.7 × 10^4^	6.5 × 10^3^ ± 1.4 × 10^4^	*p* = 0.794 U = 725.5	3.43 × 10^3^ ± 10.9 × 10^3^	8.27 × 10^3^ ± 3.0 × 10^4^	*p* = 0.558 U = 900.5
Median (IQR)	0.0 (4.0 × 10^2^)	0.0 (8.0 × 10^3^)		0.0 (3.0 × 10^2^)	0.0 (5.0 × 10^2^)	
Min–Max	0.0–1.5 × 10^5^	0.0–6.0 × 10^4^		0.0–5.0 × 10^4^	0.0–1.5 × 10^5^	
**Total coliforms (MPN/g)**						
Mean ± SD	6.2 ± 16.1	99.1 ± 3.15 × 10^2^	*p* = 0.045* U = 809.0	3.0 ± 0.0	50.1 ± 2.14 × 10^2^	*p* = 0.011* U = 990.0
Median (IQR)	3.0 (0.0)	3.0 (0.0)		0.3 (0.0)	0.3 (0.0)	
Min–Max	3.0–93.0	3.0–1.1 × 10^3^		0.3	3.0–1.1 × 10^3^	

p* significant at level < 0.05

*U* Mann–Whitney test; nonparametric used to compare quantitative variables in two groups, *IQR* Interquartile range, *TPC* Total plate count, *TC* Total coliform count, *CFU/g* Colony forming unit/gm, *MPN/g* Most probable number/gm, *SD* Standard deviation

The presence of *S. aureus* was significantly associated with the display of bread outside the shop (25.9% *versus* 11.8%) and with the non-use of gloves (27.5% *versus* 9.1%). Also, a statistically significant difference was found as regards the type of bread and the presence of *S. aureus* where the highest percentage was seen for flatbread (30.4%). Although the presence of *S. aureus* was higher among covered bread (24.6%) and unpacked bread (20.2%), the difference wasn’t statistically significant (Table 6).

**Table 6:** Distribution of *S. aureus* in the bread according to packaging, display of bread, coverage, gloves usage for unpacked bread and bread type

S. aureus	Packaging						*p* value
	**Packed**				**Unpacked**		
	**N**		**%**		**N**	**%**	
Present	11		13.1		17	20.2	$x$ ^2^ = 1.543 *p* = 0.214
Absent	73		86.9		67	79.8	
**S. aureus**	**Display of bread**						
	**Inside the shop**				**Outside the shop**		
	**N**		**%**		**N**	**%**	$x$ ^2^ = 5.393 *p* = 0.020*
Present	13		11.8		15	25.9	
Absent	97		88.2		43	74.1	
**S. aureus**	**Coverage**						
	**Covered**				**Uncovered**		
	**N**		**%**		**N**	**%**	$x$ ^2^ = 2.614 *p* = 0.106
Present	15		24.6		2	8.7	
Absent	46		75.4		21	91.3	
**S. aureus**	**Gloves usage for unpacked bread**						
	**Yes**				**No**		
	**N**		**%**		**N**	**%**	$x$ ^2^ = 4.184 *p* = 0.041*
Present	3		9.1		14	27.5	
Absent	30		90.9		37	72.5	
**S. aureus**	**Bread type**						
	**Flat**		**Fino**		**Toast**		
	**N**	**%**	**N**	**%**	**N**	**%**	$x$ ^2^ = 11.773 *p* = 0.003*
Present	17	30.4	4	7.4	**7**	12.1	
Absent	39	69.6	50	92.6	51	87.9	

p* significant at level < 0.05

$x$^2^ Chi square test used to compare qualitative variables

*S. aureus Staphylococcus aureus*

## Discussion

The results of the present work showed that while the mean TPC of the studied bread samples in both districts was higher than the accepted limit, both TC and total yeasts and moulds counts were within the permissible limits. In the same line, from different bakery products, Das et al., (2020) [15] in Bangladesh, found that bakery products were contaminated with heterotrophic bacteria within the range of 10^4^ to 10^7^ CFU/g. All the bakery items were found to harbour fungi within the acceptable microbial limits (ranging between 10^2^ and 10^3^ CFU/g).

In the current study, the mean of all counts recorded in this study (TPC, yeasts and moulds and TC) was higher among uncovered bread samples, those handled without gloves and those displayed outside the shop than their counterparts. Similar results were reported in Pakistan [16] and Bangladesh [17].

In accordance with the results of this study, a recent study conducted by Marutha and Chelule (2020) [18] concluded that selling uncovered food in open space exposed food to microbes, heat, and dust, which compromised food safety. A high frequency of contamination in packed and unpacked bread may be attributed to ineffective and inefficient packaging of products. Also, local bakeries depend on manual or hand wrapping for the packaging of bakery products which put the products at great risk of microbial contamination from the hands of packaging employees [12]. The presence of coliform organisms in food depicts a deplorable state of poor hygiene and sanitary practices employed in the processing and packaging of this food product [19].

In the present study, flatbread showed the highest level of contamination compared to Fino bread and toast in terms of TPC, total yeast and moulds as well as TC. This can be explained by less hygienic measures adopted: flatbread was displayed outside the shop, mostly uncovered and handled without gloves. Similarly, Riba et al., (2010) [20] concluded that the microbial contamination of bread samples may occur after the baking procedure of bread during hand manipulation and cutting, or as a result of keeping it in unfavorable conditions.

In contrast, Sami et al., (2020) [21] found that flour, dough and bread samples collected from bulk bread had higher counts of total bacteria than those of flatbread. However, samples collected from flatbread had higher counts of moulds. The total bacterial and moulds counts in studied bread samples were lower than the standards announced by the Institute of Standards and Industrial Research of Iran.

In the present work, the frequency of bakery workers using gloves was lower than those not using gloves during bread handling (39.3% *versus* 60.7%, respectively). Similarly, a descriptive cross-sectional study conducted by Olusegun et al., (2015) [22] in Nigeria, reported that among the bakery workers, 45% were wearing hand gloves regularly while 15% were using them occasionally. Interestingly, 45% did not see any reason why they should use gloves. This may be attributed to a lack of awareness of the importance of glove usage during handling ready-made food and finished bread.

In the current study, using gloves for the unpacked bread was accompanied by a lower mean TPC and yeasts and moulds than that without using gloves with no statistically significant difference. Also, the mean of TC was significantly lower when using gloves than without using gloves. Our results emphasized the importance of using gloves during bread handling.

Comparing the two districts involved in the present investigation, the means of TPC and total yeasts and moulds were significantly higher among bread samples collected from Al Montaza District compared to Sharq District. Also, the frequency of detection of *S. aureus* was higher among bread samples collected from Al Montaza. These results may be explained by a higher percentage of unpacked bread displayed outside the shop and handled without gloves in Al Montaza District.

In the present study, there was a statistically significant difference between the different bread locations in terms of the presence of *S. aureus*. Lack of aseptic handling and personal hygiene can result in the contamination of bread and flour with such bacteria. However, their incidence is not always regarded as hazardous to consumers; as only *S. aureus* producing enterotoxin may cause food-borne diseases [16]. In a study conducted in Nigeria, Daniyan and Nwokwu (2011) [23] observed *S. aureus* count in different stages of bread production. After baking they found 2.20×104 CFU/g S. aureus in bread samples. It might have been introduced by the handler being a normal flora on human skin.

Data from the present study revealed that there was contamination in both packed and unpacked bread which may pose a hazard to consumers. Unacceptable levels of bacteria reported in this study may be due to poor hygienic practices, dirty equipment, and polluted environments of processing and storage areas. Better manufacturing and packaging initiatives can be introduced to avoid related food safety concerns in the future. The microbiological quality of bread must be enhanced to provide safe food and better health to consumers.

### Limitations

We did not assess the enterotoxin production by the detected *S. aureus*. We did not follow the microbial counts over time.

## Conclusion

Lack of coverage and packaging of bread as well as displaying outside the shops and handling it without gloves were associated with significantly higher TPC, total yeasts and moulds and TC counts. In addition, the presence of *S. aureus* was significantly associated with displaying bread outside the shop and with the non-use of gloves. Health education is recommended to ensure the proper handling of bakery products. It is also recommended to set Egyptian Guidelines for the microbiological quality of bakery products. The formal authorities should define and clarify standards and rules on bread safety. Further studies are required to assess counts of *S. aureus* capable of enterotoxin production in bread products. Further studies are recommended for monitoring the presence of antimicrobial resistance genes in these products.

## Data Availability

The datasets used and/or analyzed during the current study are available from the corresponding author on reasonable request.

## Abbreviations

TC: Total coliform count
TPC: Total plate count
S. aureus: Staphylococcus aureus
CFU: Colony forming unit
PW: Peptone water
MPN: Most probable number
LST: Lauryl sulfate tryptose broth
BGLBB: Brilliant green lactose bile broth
